# Learning from the aircraft cockpit: optimizing anesthesia workspace layout within the primary field of view

**DOI:** 10.1186/s40981-025-00814-8

**Published:** 2025-09-26

**Authors:** Keisuke Yoshida, Yui Akama, Satoki Inoue

**Affiliations:** 1 Department of Anesthesiology, Fukushima Medical University School of Medicine, 1 Hikariga-Oka, Fukushima City, Fukushima, 960-1295 Japan; 2 Department of Anesthesiology, Takeda General Hospital, Aizuwakamatsu, Fukushima Japan

To the Editor,

The phases of general anesthesia (induction, maintenance, and emergence) are often compared to those of flight in aviation (takeoff, cruise, and landing) [1]. Just as pilots in a cockpit integrate information from multiple flight instruments to ensure passenger safety, anesthesiologists at the anesthesia workstation must manage a wide range of inputs, including anesthesia machines, ventilators, physiological monitors, the surgical field, and the movements of operating room personnel, to ensure patient safety.

Several studies have investigated the design of effective anesthesia workspaces. However, few have addressed the spatial arrangement of equipment from the perspective of visual ergonomics. We propose that the concept of the primary field of view may serve as a key element for enhancing workspace efficiency. With advances in physiological monitoring technologies and electronic medical records (EMRs), anesthesiologists are now required to process an ever-increasing volume of information both rapidly and accurately.

To reduce the workload of anesthesiologists and improve task flow efficiency, it has been suggested that EMR displays be positioned adjacent to the anesthesia machine [2]. Additionally, placing the anesthesia machine at the head of the patient—an arrangement already adopted in many institutions—may reduce workflow disruptions and enhance efficiency [3]. Although existing guidelines on ergonomics in anesthesia environments [4] emphasize the importance of promoting safety, clinical performance, and provider well-being (such as the prevention of back pain), they offer limited guidance on the optimal spatial configuration of devices and displays.

In this context, the Human Factors Design Standard published by the United States Federal Aviation Administration (FAA) [5] provides a valuable model. The guideline states that critical instruments and alerts in an aircraft cockpit be located within the pilot’s primary field of view, defined as ± 15 degrees horizontally and vertically (the “optimum visual zone”), and ideally within a maximum range of ± 35 degrees. This arrangement helps minimize eye and head movement, and we believe that this principle is directly applicable to the anesthesia workspace. Key elements that require the anesthesiologist’s attention should ideally be positioned within the narrowest possible viewing angle. For example, the placement of a mobile anesthesia machine and the anesthesiologist’s position can be easily adjusted to conform to this principle (Figs. 1, 2). While institutional limitations and individual preferences must be considered, applying this “narrow viewing angle” framework can help create an efficient and ergonomic anesthesia workspace at no additional cost and with minimal effort.

**Fig. 1:**
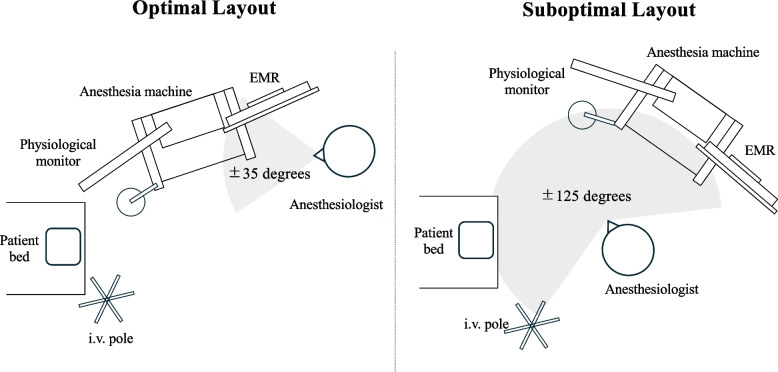
Examples of optimal and suboptimal positioning of the anesthesia machine relative to the patient bed. In the optimal configuration, all key elements required for anesthetic management are located within ± 35 degrees of the anesthesiologist’s line of sight. In contrast, the example of a suboptimal layout may require the anesthesiologist to turn frequently, as essential information is dispersed over a viewing angle exceeding ± 125 degrees. EMR: electronic medical record

**Fig. 2:**
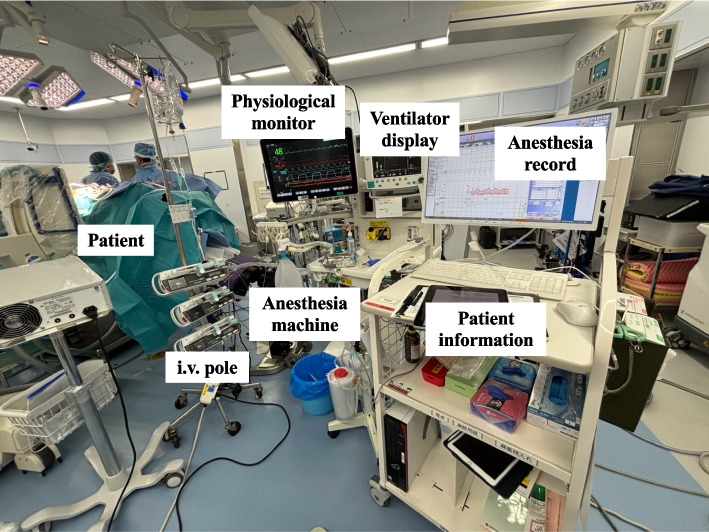
View from the anesthesiologist’s perspective during spinal surgery (an optimal example). Although the anesthesiologist could not be positioned directly at the head of the patient in the prone position due to equipment layout constraints, all relevant information required for anesthetic management was located within approximately ± 30 degrees. The displays of the syringe pumps mounted on the intravenous pole were also oriented toward the anesthesiologist

On the other hand, the environment of anesthesiologists is less standardized than that of pilots. While aircraft cockpits follow uniform ergonomic designs, anesthesia machines and monitors differ among manufacturers, and operating rooms vary widely across institutions and even from case to case. In addition, unlike pilots who remain seated, anesthesiologists must often move around the operating room for tasks such as vascular access, drug preparation, or transesophageal echocardiography. Thus, it is important for anesthesiologists to cultivate the habit of creating an efficient workspace suited to each situation, while incorporating the principle of a relatively narrow range of eye movement. Ultimately, fostering this habit can contribute to a safer, more efficient, and less stressful working environment for anesthesiologists, thereby enhancing both patient care and provider well-being.

## Data Availability

Not applicable.
